# Approximation properties of Chlodowsky variant of $(p,q)$ Bernstein-Stancu-Schurer operators

**DOI:** 10.1186/s13660-017-1451-7

**Published:** 2017-08-01

**Authors:** Vishnu Narayan Mishra, M Mursaleen, Shikha Pandey, Abdullah Alotaibi

**Affiliations:** 1 Department of Mathematics, Indira Gandhi National Tribal University, Lalpur, Amarkantak, Anuppur, Madhya Pradesh 484887 India; 2 Department of Mathematics, Aligarh Muslim University, Aligarh, 202002 India; 3 Department of Applied Mathematics & Humanities, Sardar Vallabhbhai National Institute of Technology, Surat, Gujarat 395007 India; 4 Operator Theory and Applications Research Group, Department of Mathematics, Faculty of Science, King Abdulaziz University, Jeddah, 21589 Saudi Arabia; 5 L. 1627 Awadh Puri Colony, Phase-III, Beniganj, Opposite-Industrial Training Institute (I.T.I.), Ayodhya Main Road, Faizabad, Uttar Pradesh 224001 India

**Keywords:** 41A25, 41A36, 41A10, 41A30, $(p,q)$-integers, $(p,q)$-Bernstein operators, linear positive operators

## Abstract

In the present paper, we introduce the Chlodowsky variant of $(p,q)$ Bernstein-Stancu-Schurer operators which is a generalization of $(p,q)$ Bernstein-Stancu-Schurer operators. We also discuss its Korovkin-type approximation properties and rate of convergence.

## Introduction and preliminaries

In 1912, Bernstein [1] introduced the following sequence of operators $B_{n} : C[0, 1] \rightarrow C[0, 1]$ defined for any $n \in \mathbb{N}$ and for any function $f \in C[0, 1]$:

1.1 $$ B_{n}(f;x)=\sum_{k=0}^{n} \left ( \textstyle\begin{array}{c} n \\ k \end{array}\displaystyle \right ) x^{k} (1-x)^{n-k} f \biggl(\frac{k}{n} \biggr), \quad x\in[0,1]. $$

Later various generalizations of these operators were discovered. It has been proved as a powerful tool for numerical analysis, computer aided geometric design and solutions of differential equations. In last two decades, the applications of *q*-calculus has played an important role in the area of approximation theory, number theory and theoretical physics. In 1987, Lupaş [2] and in 1997, Phillips [3] introduced a sequence of Bernstein polynomials based on *q*-integers and investigated its approximation properties. Several researchers obtained various other generalizations of operators based on *q*-calculus. For any function $f \in C[0, 1]$ the *q*-form of Bernstein operator is described by Lupaş [2] as

1.2 $$ L_{n,q}(f;x)=\sum_{k=0}^{n} \frac{\left [ {\scriptsize\begin{matrix}{}n \cr k\end{matrix}} \right ]_{q} q^{k(k-1)/2}x^{k} (1-x)^{n-k}}{\prod_{j=0}^{n-1}(1-x+q^{j}x)}f \biggl(\frac{[k]_{q}}{[n]_{q}} \biggr), \quad x\in[0,1]. $$

In 1932, Chlodowsky [4] presented a generalization of Bernstein polynomials on an unbounded set, known as Bernstein-Chlodowsky polynomials,

1.3 $$ B_{n}(f,x)=\sum_{k=0}^{n} f \biggl(\frac{k}{n}b_{n} \biggr)\left ( \textstyle\begin{array}{c} n \\ k \end{array}\displaystyle \right ) \biggl(\frac{x}{b_{n}} \biggr)^{k} \biggl(1- \frac{x}{b_{n}} \biggr)^{n-k},\quad 0\leq x\leq b_{n}, $$

where $b_{n}$ is an increasing sequence of positive terms with the properties $b_{n} \rightarrow\infty$ and $\frac{b_{n}}{n} \rightarrow0$ as $n \rightarrow\infty$.

In 2008, Karsli and Gupta [5] expressed the *q*-analogue of Bernstein-Chlodowsky polynomials by

1.4 $$ C_{n}(f;q;x)=\sum_{k=0}^{n} \left [ \textstyle\begin{array}{c} n \\ k \end{array}\displaystyle \right ]_{q} \biggl( \frac{x}{b_{n}} \biggr)^{k} \prod_{s=0}^{n-k-1} \biggl(1-q^{s} \frac{x}{b_{n}} \biggr)f \biggl(\frac{[k]_{q}}{[n]_{q}}b_{n} \biggr),\quad 0\leq x\leq b_{n}, $$

where $b_{n}$ is an increasing sequence of positive values, with the properties $b_{n} \rightarrow\infty$ and $\frac{b_{n}}{[n]_{q}} \rightarrow 0$ as $n \rightarrow\infty$.

Recently, Mursaleen *et al.* [6–9] proposed and analyzed approximation properties for $(p,q)$ analogue of Bernstein operators, Bernstein-Stancu operators and Bernstein-Schurer operators. Besides this, we also refer to some recent related work on this topic: *e.g.* [10–20].

In 2015, Mursleen *et al.* [7], investigated the $(p,q)$ form of the Bernstein-Stancu operator, which is given by

1.5 $$ S_{n}^{(\alpha,\beta)}(f;x,p,q)=\frac{1}{p^{\frac{n(n-1)}{2}}}\sum _{k=0}^{n} \left [ \textstyle\begin{array}{c} n \\ k \end{array}\displaystyle \right ]_{p,q}p^{\frac{k(k-1)}{2}} x^{k} \prod _{s=0}^{n-k-1} \bigl(p^{s}-q^{s}x \bigr) f \biggl(\frac{p^{n-k}[k]_{p,q}+\alpha}{[n]_{p,q}+\beta} \biggr), $$

where $\alpha, \beta$ are non-negative integers and $f \in C[0, 1]$, $x \in[0,1]$ and $0 \leq\alpha\leq\beta$.

For the first few moments, we get the following lemma.

### Lemma 1

See [7]

*For the operators* $S_{n}^{(\alpha,\beta)}$, *we have*

1. $S_{n}^{(\alpha,\beta)}(1;x,p,q)=1$,
2. $S_{n}^{(\alpha,\beta)}(t;x,p,q)=\frac{[n]_{p,q}x+\alpha }{[n]_{p,q}+\beta}$,
3. $S_{n}^{(\alpha,\beta)}(t^{2};x,p,q)=\frac{1}{([n]_{p,q}+\beta )^{2}}(q[n]_{p,q}[n-1]_{p,q}x^{2}+[n]_{p,q}(2\alpha+ p^{n-1})x+\alpha^{2})$.

## Construction of the operators

Considering the revised form of $(p,q)$ analogue of Bernstein operators [7], we construct the Chlodowsky variant of $(p,q)$ Bernstein-Stancu-Schurer operators as

2.1 $$ \begin{aligned}[b]C_{n,m}^{(\alpha,\beta)}(f;x,p,q)={}&\frac{1}{p^{\frac{(n+m)(n+m-1)}{2}}}\sum _{k=0}^{n+m} \left [ \textstyle\begin{array}{c} n+m \\ k \end{array}\displaystyle \right ]_{p,q} p^{\frac{k(k-1)}{2}} \biggl( \frac{x}{b_{n}} \biggr)^{k}\\&\times \prod_{s=0}^{n+m-k-1} \biggl(p^{s}-q^{s}\frac{x}{b_{n}} \biggr) f \biggl( \frac {p^{n+m-k}[k]_{p,q}+\alpha}{[n]_{p,q}+\beta}b_{n} \biggr), \end{aligned}$$

where $n\in\mathbb{N}$, $m,\alpha,\beta\in\mathbb{N}_{0}$, with $\frac {\alpha}{\beta} \approx1$, $0 \leq x \leq b_{n}$, $0< q < p\leq1 $ and $b_{n}$ is an increasing sequence of positive terms with the properties $b_{n} \rightarrow\infty$ and $\frac{b_{n}}{[n]_{p,q}} \to0$ as $n \to\infty$. Evidently, $C_{n,m}^{(\alpha,\beta)}$ is a linear and positive operator. Consider the case if $p,q \rightarrow1$ and $m= 0$ in (2.1), then it will reduce to the Stancu-Chlodowsky polynomials [21].

Let us assume the number $n+m=n_{m}$, we will use this notation throughout in this paper. Next, we have obtained the following lemma using simple calculations.

### Lemma 2

*Let* $C_{n,m}^{(\alpha,\beta)}(f;x,p,q)$ *be given by* (2.1). *The first few moments of the operators are*

(i) $C_{n,m}^{(\alpha,\beta)}(1;x,p,q)=1$,
(ii) $C_{n,m}^{(\alpha,\beta)}(t;x,p,q)=\frac {[n_{m}]_{p,q}x+\alpha b_{n}}{[n]_{p,q}+\beta}$,
(iii) $C_{n,m}^{(\alpha,\beta)}(t^{2};x,p,q)=\frac {1}{([n]_{p,q}+\beta )^{2}}(q[n_{m}]_{p,q}[n_{m}-1]_{p,q}x^{2}+[n_{m}]_{p,q}(2\alpha+p^{n_{m}-1})b_{n} x+\alpha^{2} b_{n}^{2})$,
(iv) $C_{n,m}^{(\alpha,\beta)}((t-x);x,p,q)= (\frac {[n_{m}]_{p,q}}{[n]_{p,q}+\beta}-1 )x+\frac{\alpha b_{n}}{[n]_{p,q}+\beta}$,
(v) $$\begin{aligned} C_{n,m}^{(\alpha,\beta)}\bigl((t-x)^{2};x,p,q\bigr) ={}& \biggl(1-2\frac {[n_{m}]_{p,q}}{([n]_{p,q}+\beta)}+\frac {q[n_{m}]_{p,q}[n_{m}-1]_{p,q}}{([n]_{p,q}+\beta)^{2}} \biggr)x^{2} \\ &+ \biggl( \frac{(2\alpha+p^{n_{m}-1})[n_{m}]_{p,q}}{([n]_{p,q}+\beta )}-2\alpha \biggr) \frac{b_{n}}{([n]_{p,q}+\beta)} x\\ & + \frac{\alpha^{2} b_{n}^{2}}{([n]_{p,q}+\beta)^{2}}. \end{aligned}$$

### Proof

(i)

$$C_{n,m}^{(\alpha,\beta)}(1;x,p,q)=\frac{1}{p^{\frac{(n_{m})(n_{m}-1)}{2}}}\sum _{k=0}^{n_{m}} \left [ \textstyle\begin{array}{c} n_{m} \\ k \end{array}\displaystyle \right ]_{p,q} p^{\frac{k(k-1)}{2}} \biggl(\frac{x}{b_{n}} \biggr)^{k} \prod_{s=0}^{n_{m}-k-1} \biggl(p^{s}-q^{s}\frac{x}{b_{n}} \biggr)=1. $$

(ii)

$$ \begin{aligned} C_{n,m}^{(\alpha,\beta)}(t;x,p,q)={}&\frac{1}{p^{\frac{n_{m}(n_{m}-1)}{2}}}\sum _{k=0}^{n_{m}} \left [ \textstyle\begin{array}{c} n_{m} \\ k \end{array}\displaystyle \right ]_{p,q} p^{\frac{k(k-1)}{2}} \biggl(\frac{x}{b_{n}} \biggr)^{k} \\ &\times\prod_{s=0}^{(n_{m}-k-1)} \biggl(p^{s}-q^{s}\frac{x}{b_{n}} \biggr) \biggl( \frac {p^{n_{m}-k}[k]_{p,q}+\alpha}{[n]_{p,q}+\beta}b_{n} \biggr) \\ ={}& \frac{[n_{m}]_{p,q}}{p^{\frac{n_{m}(n_{m}-3)}{2}}([n]_{p,q}+\beta)}\sum_{k=0}^{n_{m}-1} \left [ \textstyle\begin{array}{c} n_{m}-1 \\ k \end{array}\displaystyle \right ]_{p,q} p^{\frac{k(k+1)}{2}} \biggl(\frac{x}{b_{n}} \biggr)^{k+1}\\ &\times \prod_{s=0}^{(n_{m}-k-2)} \biggl(p^{s}-q^{s} \frac{x}{b_{n}} \biggr) \biggl(\frac {b_{n}}{p^{k+1}} \biggr) \\ & + \frac{1}{p^{\frac{n_{m}(n_{m}-1)}{2}}}\sum_{k=0}^{n_{m}} \left [ \textstyle\begin{array}{c} n_{m} \\ k \end{array}\displaystyle \right ]_{p,q} p^{\frac{k(k-1)}{2}} \biggl(\frac{x}{b_{n}} \biggr)^{k}\\ &\times \prod _{s=0}^{(n_{m}-k-1)} \biggl(p^{s}-q^{s} \frac{x}{b_{n}} \biggr) \biggl(\frac {\alpha}{[n]_{p,q}+\beta}b_{n} \biggr) \\ ={}& \frac{[n_{m}]_{p,q}}{([n]_{p,q}+\beta)}x \frac{1}{p^{\frac{(n_{m}-1)(n_{m}-2)}{2}}}\sum_{k=0}^{n_{m}-1} \left [ \textstyle\begin{array}{c} n_{m}-1 \\ k \end{array}\displaystyle \right ]_{p,q} p^{\frac{k(k-1)}{2}} \biggl(\frac{x}{b_{n}} \biggr)^{k} \\ &\times\prod _{s=0}^{n_{m}-k-2} \biggl(p^{s}-q^{s} \frac{x}{b_{n}} \biggr) + \frac{\alpha b_{n} }{([n]_{p,q}+\beta)} \\ = {}&\frac{[n_{m}]_{p,q}}{([n]_{p,q}+\beta)}x+\frac{\alpha b_{n} }{([n]_{p,q}+\beta)}. \end{aligned}$$

(iii)

$$\begin{aligned} C_{n,m}^{(\alpha,\beta)}\bigl(t^{2};x,p,q\bigr)={}& \frac{1}{p^{\frac{n_{m}(n_{m}-1)}{2}}}\sum_{k=0}^{n_{m}} \left [ \textstyle\begin{array}{c} n_{m} \\ k \end{array}\displaystyle \right ]_{p,q} p^{\frac{k(k-1)}{2}} \biggl(\frac{x}{b_{n}} \biggr)^{k}\\ &\times \prod _{s=0}^{(n_{m}-k-1)} \biggl(p^{s}-q^{s} \frac{x}{b_{n}} \biggr) \biggl(\frac {p^{n_{m}-k}[k]_{p,q}+\alpha}{[n]_{p,q}+\beta}b_{n} \biggr)^{2} \\ ={}& \frac{1}{p^{\frac{n_{m}(n_{m}-1)}{2}}}\frac{1}{([n]_{p,q}+\beta)^{2}} \Biggl[ p^{2n_{m}} \sum_{k=0}^{n_{m}} \left [ \textstyle\begin{array}{c} n_{m} \\ k \end{array}\displaystyle \right ]_{p,q} p^{\frac{k(k-1)}{2}} \biggl( \frac{x}{b_{n}} \biggr)^{k}\\ &\times\prod_{s=0}^{(n_{m}-k-1)} \biggl(p^{s}-q^{s}\frac{x}{b_{n}} \biggr) \frac {b_{n}^{2}[k]_{p,q}^{2}}{p^{2k}} \\ & + 2\alpha p^{n_{m}} \sum_{k=0}^{n_{m}} \left [ \textstyle\begin{array}{c} n_{m} \\ k \end{array}\displaystyle \right ]_{p,q} p^{\frac{k(k-1)}{2}} \biggl(\frac{x}{b_{n}} \biggr)^{k}\\ &\times \prod _{s=0}^{(n_{m}-k-1)} \biggl(p^{s}-q^{s} \frac{x}{b_{n}} \biggr) \frac {b_{n}^{2}[k]_{p,q}}{p^{k}} \\ & + \alpha^{2} \sum_{k=0}^{n_{m}} \left [ \textstyle\begin{array}{c} n_{m} \\ k \end{array}\displaystyle \right ]_{p,q} p^{\frac{k(k-1)}{2}} \biggl(\frac{x}{b_{n}} \biggr)^{k}\\ &\times \prod _{s=0}^{(n_{m}-k-1)} \biggl(p^{s}-q^{s} \frac{x}{b_{n}} \biggr)b_{n}^{2} \Biggr] \\ ={}& \frac{1}{([n]_{p,q}+\beta)^{2}} \Biggl[\frac{p^{2n_{m}}}{p^{\frac{n_{m}(n_{m}-1)}{2}}}[n_{m}]_{p,q}b_{n}^{2} \sum_{k=0}^{n_{m}-1} \left [ \textstyle\begin{array}{c} n_{m}-1 \\ k \end{array}\displaystyle \right ]_{p,q} p^{\frac{k(k+1)}{2}} \biggl( \frac{x}{b_{n}} \biggr)^{k+1}\\ &\times \prod_{s=0}^{(n_{m}-k-2)} \biggl(p^{s}-q^{s}\frac{x}{b_{n}} \biggr) \frac {[k+1]_{p,q}}{p^{2(k+1)}} \\ & + \frac{p^{n_{m}}}{p^{\frac{n_{m}(n_{m}-1)}{2}}}2\alpha[n_{m}]_{p,q} b_{n}^{2}\sum_{k=0}^{n_{m}-1} \left [ \textstyle\begin{array}{c} n_{m}-1 \\ k \end{array}\displaystyle \right ]_{p,q} p^{\frac{k(k+1)}{2}} \biggl(\frac{x}{b_{n}} \biggr)^{k+1}\\ &\times \prod_{s=0}^{(n_{m}-k-2)} \biggl(p^{s}-q^{s} \frac{x}{b_{n}} \biggr) \frac {1}{p^{(k+1)}} +\alpha^{2} b_{n}^{2} \Biggr]. \end{aligned}$$

Now using $[k+1]_{p,q}=p^{k}+q[k]_{p,q}$, we will obtain the result.

Using the linear property of operators, we have

$$\begin{aligned} C_{n,m}^{(\alpha,\beta)}\bigl((t-x);x,p,q\bigr)&=C_{n,m}^{(\alpha,\beta )}(t;x,p,q)-xC_{n,m}^{(\alpha,\beta)}(1;x,p,q) \\ &= \biggl(\frac{[n_{m}]_{p,q}}{[n]_{p,q}+\beta}-1 \biggr)x+\frac{\alpha b_{n}}{[n]_{p,q}+\beta}. \end{aligned}$$

Hence, we get (iv).

Similar calculations give

$$C_{n,m}^{(\alpha,\beta)}\bigl((t-x)^{2};x,p,q \bigr)=C_{n,m}^{(\alpha,\beta )}\bigl(t^{2};x,p,q \bigr)-2xC_{n,m}^{(\alpha,\beta)}(t;x,p,q)+x^{2}C_{n,m}^{(\alpha ,\beta)}(1;x,p,q). $$

Substituting the results of (i), (ii) and (iii), we prove the result (v). □

### Lemma 3

*For every fixed* $0< q< p \leq1$, *we have*

$$ \frac{[n_{m}]_{p,q}[n_{m}-1]_{p,q}}{([n]_{p,q}+\beta)^{2}}q-2\frac {[n_{m}]_{p,q}}{[n]_{p,q}+\beta}+1 \leq \biggl(\frac {(p^{n}+q^{n})[m]_{p,q}-\beta}{[n]_{p,q}+\beta} \biggr)^{2}. $$

### Proof

$$\begin{aligned}{} [n_{m}-1]_{p,q}q = &\frac{p^{n+m-1}-q^{n+m-1}}{p-q}q \\ = &\frac{p^{n+m-1}q-q^{n+m}}{p-q} \\ \leq& \frac{p^{n+m}-q^{n+m}}{p-q} \quad\bigl(\text{since }q< p \Rightarrow p^{n+m-1}q< p^{n+m}\bigr) \\ = &[n_{m}]_{p,q}. \end{aligned}$$

Thus, $[n_{m}]_{p,q}[n_{m}-1]_{p,q} q \leq[n_{m}]_{p,q}^{2}$, and we get

$$ \begin{gathered} \frac{[n_{m}]_{p,q}[n_{m}-1]_{p,q}}{([n]_{p,q}+\beta)^{2}}q-2\frac {[n_{m}]_{p,q}}{[n]_{p,q}+\beta}+1\\ \quad \leq \biggl(\frac{[n_{m}]_{p,q}}{[n]_{p,q}+\beta}-1 \biggr)^{2} \\ \quad = \frac{1}{([n]_{p,q}+\beta)^{2}} \biggl\{ \frac {p^{n+m}-q^{n+m}}{p-q}-\frac{p^{n}-q^{n}}{p-q}-\beta \biggr\} ^{2} \\ \quad = \frac{1}{([n]_{p,q}+\beta)^{2}} \biggl\{ \frac{p^{n} p^{m}-q^{n} q^{m}-p^{n}+q^{n}}{p-q}-\beta \biggr\} ^{2} \\ \quad = \frac{1}{([n]_{p,q}+\beta)^{2}} \biggl\{ \frac{p^{n} p^{m}-p^{n}q^{m}+p^{m}q^{n}-q^{n} q^{m}+p^{n}q^{m}-p^{n}+q^{n}-p^{m}q^{n}}{p-q}-\beta \biggr\} ^{2} \\ \quad = \frac{1}{([n]_{p,q}+\beta)^{2}} \biggl\{ \frac{p^{n} (p^{m}-q^{m})+q^{n} (p^{m}- q^{m})+p^{n}(q^{m}-1)+q^{n}(1-p^{m})}{p-q}-\beta \biggr\} ^{2} \\ \quad = \frac{1}{([n]_{p,q}+\beta)^{2}} \biggl\{ \frac {(p^{n}+q^{n})(p^{m}-q^{m})+q^{n}(1-p^{m})-p^{n}(1-q^{m})}{p-q}-\beta \biggr\} ^{2} \\ \quad = \frac{1}{([n]_{p,q}+\beta)^{2}} \biggl\{ \bigl(p^{n}+q^{n} \bigr)[m]_{p,q}-\beta- \frac{p^{n}(1-q^{m})-q^{n}(1-p^{m})}{p-q} \biggr\} ^{2} \\ \quad \leq \frac{((p^{n}+q^{n})[m]_{p,q}-\beta )^{2}}{([n]_{p,q}+\beta)^{2}} \quad\text{since } 0 < q < p \leq1. \end{gathered}$$

We can conclude the last inequality using the following statements:

Since $0 < q < p \leq1$, we have $0 < q^{n} < p^{n} \leq1$ and $0<(1-p^{m})<(1-q^{m}) \leq1$, hence $q^{n}(1-p^{m})< p^{n}(1-q^{m})$ *i.e.* $p^{n}(1-q^{m})-q^{n}(1-p^{m})>0$. □

### Remark 1

As a result of Lemma 2 and 3, we have

$$\begin{aligned} C_{n,m}^{(\alpha,\beta)}\bigl((t-x)^{2};x,p,q\bigr)\leq{}& \biggl( \frac {((p^{n}+q^{n})[m]_{p,q}-\beta)^{2}}{([n]_{p,q}+\beta)^{2}} \biggr)x^{2} + \biggl( \frac{[n_{m}]_{p,q}(2\alpha+p^{n-1})}{([n]_{p,q}+\beta)^{2}} \biggr) {b_{n}x} \\ & +\frac{\alpha^{2} b_{n}^{2}}{([n]_{p,q}+\beta)^{2}}. \end{aligned}$$

## Results and discussion

In this paper we have constructed and investigated a Chlodowsky variant of $(p,q)$ Bernstein-Stancu-Schurer operator. We have showed that our modified operators have a better error estimation than the classical ones. We have also obtained some approximation results with the help of the well-known Korovkin theorem and the weighted Korovkin theorem for these operators. Furthermore, we studied convergence properties in terms of the modulus of continuity for functions in Lipschitz class. Next we have also obtained the Voronovskaja-type result for these operators.

### Korovkin-type approximation theorem

Assume $C_{\rho}$ is the space of all continuous functions *f* such that

$$\big|f(x)\big| \leq M \rho(x),\quad a< x< b, $$

and $\rho(x)$ is the weight function.

Then $C_{\rho}$ is a Banach space with the norm

$$\|f\|_{\rho}=\sup_{a< x< b}\frac{|f(x)|}{\rho(x)}. $$

Consider the subspace $C_{\rho}^{0} := \{ f \in C_{\rho}: \lim_{|x| \to\infty} \frac{|f(x)|}{\rho(x)}\text{ is finite}\}$.

The subsequent Theorem 1 is a Korovkin approximation theorem in weighted space.

#### Theorem 1

See [22]

*There exists a sequence of positive linear operators* $U_{n}$, *acting from* $C_{\rho}^{0}$ *to* $C_{\rho}^{0} $, *satisfying the conditions*

1. $\lim_{n\rightarrow\infty}\|U_{n}(1;\cdot)-1\|_{\rho}=0$,
2. $\lim_{n\rightarrow\infty}\|U_{n}(\phi;\cdot)-\phi\| _{\rho}=0$,
3. $\lim_{n\rightarrow\infty}\|U_{n}(\phi^{2};\cdot)-\phi^{2}\| _{\rho}=0$,

*where* $\phi(x)$ *is a continuous and increasing function on* $(-\infty ,\infty)$ *such that* $\lim_{x\to\pm\infty} \phi(x) = \pm\infty $ *and* $\rho(x) = 1 + \phi^{2}$, *and there exists a function* $f^{*} \in C_{\rho}^{0}$ *for which*

$$\lim_{n\to\infty}\|U_{n}f-f\|_{\rho}=0. $$

Consider the weight function $\rho(x)=1+x^{2}$ and operator (see [23])

$$U_{n,m}^{\alpha,\beta}(f;x,p,q)= \textstyle\begin{cases} C_{n,m}^{\alpha,\beta}(f;x,p,q)&\text{if } x \in[0,b_{n}],\\ f(x)& \text{if } x \notin [0,b_{n}]. \end{cases} $$

For $f \in C_{1+x^{2}}$, we have

$$\begin{aligned} \big\| U_{n,m}^{\alpha,\beta}(f;\cdot,p,q)\big\| _{1+x^{2}} &\leq\sup _{x\in [0,b_{n}]} \frac{|C_{n,m}^{\alpha,\beta}(f;x,p,q)|}{1+x^{2}} +\sup_{x\in (b_{n},\infty)} \frac{|f(x)|}{1+x^{2}} \\ & \leq\|f\|_{1+x^{2}} \biggl[ \sup_{x\in[0,b_{n})} \frac{|C_{n,m}^{\alpha ,\beta}(1+t^{2};x,p,q)|}{1+x^{2}}+1 \biggr]. \end{aligned}$$

Now, using Lemma 2 we will obtain

3.1 $$ \big\| U_{n,m}^{\alpha,\beta}(f;\cdot,p,q)\big\| _{1+x^{2}} \leq M \|f \|_{1+x^{2}}, $$

which means that $U_{n,m}^{\alpha,\beta}(f;\cdot,p,q)$ is bounded operator, henceforth a continuous operator too. Since ‘An operator between two normed spaces is a bounded linear operator if and only if it is a continuous linear operator.’

Now, consider the sequences $(p_{n})$ and $(q_{n})$ for $0< q_{n}< p_{n}\leq1$ satisfying

3.2 $$ \begin{gathered}\lim_{n \to\infty} p_{n}=\lim _{n \to\infty} q_{n}=1,\\ \lim_{n \to\infty} p^{n}_{n}=a,\qquad\lim_{n \to\infty} q^{n}_{n}=c\quad \text{where }0< a,c< 1,a \neq c \text{ hence }\lim_{n\to\infty}[n]_{p_{n},q_{n}}=\infty. \end{gathered}$$

#### Theorem 2

*For all* $f\in C_{1+x^{2}}^{0}$, $0\leq x \leq b_{n}$, *we have*

3.3 $$ \lim_{n\to\infty}\big\| U_{n,m}^{\alpha,\beta}(f; \cdot ,p_{n},q_{n})-f(\cdot)\big\| _{1+x^{2}}=0 $$

*provided that* $p:=(p)_{n}$, $q:=(q)_{n}$ *with* $0< q_{n} < p_{n}\leq1$ *satisfying* (3.2) *and* $\lim_{n\to\infty}\frac{b_{n}}{[n]_{p_{n},q_{n}}}=0$.

#### Proof

Using the results of Theorem 1 and Lemma 2(i), (ii) and (iii), we will obtain the following assessments, respectively:

3.4 $$\begin{aligned}& \sup_{ 0\leq x \leq b_{n}}\frac{|U_{n,m}^{\alpha,\beta }(1;x,p_{n},q_{n})-1|}{1+x^{2}}=0, \end{aligned}$$

3.5 $$\begin{aligned}& \begin{aligned}[b]\sup_{ 0\leq x \leq b_{n}}\frac{|U_{n,m}^{\alpha,\beta }(t;x,p_{n},q_{n})-x|}{1+x^{2}} &\leq \sup _{0\leq x \leq b_{n}}\frac{ \vert \frac{[n_{m}]_{p_{n},q_{n}}}{[n]_{p_{n},q_{n}}+\beta}-1 \vert x+\frac{\alpha b_{n}}{[n]_{p_{n},q_{n}}+\beta}}{1+x^{2}} \\ &\leq \biggl\vert \frac{[n_{m}]_{p_{n},q_{n}}}{[n]_{p_{n},q_{n}}+\beta}-1 \biggr\vert +\frac{\alpha b_{n}}{[n]_{p_{n},q_{n}}+\beta} \rightarrow0, \end{aligned} \end{aligned}$$

and

3.6 $$\begin{aligned} &\sup_{0\leq x \leq b_{n}}\frac{|U_{n,m}^{\alpha,\beta }(t^{2};x,p_{n},q_{n})-x^{2}|}{1+x^{2}} \\ &\quad\leq \sup_{ 0\leq x \leq b_{n}}\frac{1}{1+x^{2}} \bigg|\frac {(q_{n}[n_{m}]_{p_{n},q_{n}}[n_{m}-1]_{p_{n},q_{n}}x^{2} +[n_{m}]_{p_{n},q_{n}}(2\alpha +p_{n}^{n_{m}-1})b_{n} x+\alpha^{2} b_{n}^{2})}{([n]_{p_{n},q_{n}}+\beta)^{2}}-x^{2} \bigg| \\ &\quad \leq \biggl\{ \bigg| \frac {q_{n}[n_{m}]_{p_{n},q_{n}}[n_{m}-1]_{p_{n},q_{n}}}{([n]_{p_{n},q_{n}}+\beta)^{2}}-1 \bigg|+ \bigg|\frac{[n_{m}]_{p_{n},q_{n}}(2\alpha +p_{n}^{n_{m}-1})}{([n]_{p_{n},q_{n}}+\beta)^{2}} \bigg| \frac{b_{n}}{2}+\frac{\alpha ^{2}}{([n]_{p_{n},q_{n}}+\beta)^{2}} \biggr\} \\ &\quad \to 0, \end{aligned}$$

whenever $n \to\infty$.

Since the weight function is invariant w.r.t. positive and negative values of *x*, and conditions (3.4)-(3.6) are true for all $t\in\mathbb{R}$, we can use Theorem 1 and get the desired result (3.3), which implies that the operator sequence $C_{n,m}^{\alpha,\beta}$ converges uniformly to any continuous function in weighted space $C_{1+x^{2}}^{0}$ for $x \in[0, b_{n}]$. □

#### Theorem 3

*Assuming* *c* *as a positive and real number independent of* *n* *and* *f* *as a continuous function which vanishes on* $[c,\infty)$. *Let* $p := (p_{n})$, $q := (q_{n})$ *with* $0 < q_{n} < p_{n} \leq1$ *satisfying* (3.2) *and* $\lim_{n\to\infty} \frac{b_{n}^{2}}{[n]_{p_{n},q_{n}}} =0$. *Then we have*

$$\lim_{n\to\infty}\sup_{0 \leq x\leq b_{n}} \bigl\vert C_{n,m}^{\alpha ,\beta}(f;x,p_{n},q_{n})- f(x) \bigr\vert =0. $$

#### Proof

From the hypothesis on *f*, it is bounded *i.e.* $|f(x)| \leq M$ ($M>0$). For any $\epsilon>0$, we have

$$\biggl\vert f \biggl(\frac{p_{n}^{n_{m}-k}[k]_{p_{n},q_{n}}+\alpha}{[n]_{p_{n},q_{n}}+\beta }b_{n} \biggr)-f(x) \biggr\vert < \epsilon+\frac{2M}{\delta^{2}} \biggl(\frac {p_{n}^{n_{m}-k}[k]_{p_{n},q_{n}}+\alpha}{[n]_{p_{n},q_{n}}+\beta}b_{n}-x \biggr)^{2}, $$

where $x\in[0,b_{n}]$ and $\delta=\delta(\epsilon)$ are independent of *n*. Operating with the operator (2.1) on both sides, we can conclude by using Lemma 3 and Remark 1,

$$\begin{aligned} \sup_{0 \leq x\leq b_{n}} \bigl\vert C_{n,m}^{\alpha,\beta}(f;x,p_{n},q_{n}) - f(x) \bigr\vert \leq{}&\epsilon+\frac{2M}{\delta^{2}} b_{n}^{2} \biggl\{ \bigg|\frac{((p^{n}+q^{n})[m]_{p,q}-\beta)^{2}}{([n]_{p,q}+\beta)^{2}}\bigg| \\ &+ \bigg| \frac{(2\alpha +p_{n}^{n_{m}-1})[n_{m}]_{p_{n},q_{n}}}{([n]_{p_{n},q_{n}}+\beta)^{2}} \bigg| +\frac {\alpha^{2} }{([n]_{p_{n},q_{n}}+\beta)^{2}} \biggr\} . \end{aligned}$$

Since $\frac{b_{n}^{2}}{[n]_{p_{n},q_{n}}} =0$ as $n \to\infty$, we have the desired result. □

### Rate of convergence

We will find the rate of convergence for functions in the Lipschitz class $\mathit{Lip}_{M}(\gamma)$ ($0 < \gamma\leq1$). Assume that $C_{B}[0,\infty )$ denotes the space of bounded continuous functions on $[0,\infty)$. A function $f \in C_{B}[0,\infty)$ belongs to $\mathit{Lip}_{M}(\gamma)$ if

$$\big|f (t) - f (x)\big| \leq M|t - x|^{\gamma}, \quad\text{for } t,x \in[0,\infty). $$

#### Theorem 4

*Let* $f \in \mathit{Lip}_{M}(\gamma)$, *then*

$$\big|C_{n,m}^{\alpha,\beta}(f;x,p,q)-f(x)\big|\leq M\bigl(\lambda _{n,p,q}(x)\bigr)^{\gamma/2}, $$

*where* $\lambda_{n,p,q}(x)=C_{n,m}^{\alpha,\beta}((t-x)^{2};x,p,q)$.

#### Proof

Since $f \in \mathit{Lip}_{M}(\gamma)$, and the operator $C_{n,m}^{\alpha,\beta }(f;x,p,q)$ is linear and monotone,

$$\begin{aligned} \big| C_{n,m}^{\alpha,\beta}(f;x,p,q)- f(x)\big| =& \Bigg|\frac{1}{p^{\frac{n_{m}(n_{m}-1)}{2}}}\sum _{k=0}^{n_{m}} \left [ \textstyle\begin{array}{c} n_{m} \\ k \end{array}\displaystyle \right ]_{p,q} p^{\frac{k(k-1)}{2}} \biggl( \frac{x}{b_{n}} \biggr)^{k} \\ &\quad\times \prod_{s=0}^{(n_{m}-k-1)} \biggl(p^{s}-q^{s}\frac{x}{b_{n}} \biggr)f \biggl(\frac{p^{n_{m}-k}[k]_{p,q}+\alpha}{[n]_{p,q}+\beta}b_{n} \biggr)-f(x) \Bigg| \\ \leq&\frac{1}{p^{\frac{n_{m}(n_{m}-1)}{2}}}\sum_{k=0}^{n_{m}} \left [ \textstyle\begin{array}{c} n_{m} \\ k \end{array}\displaystyle \right ]_{p,q} p^{\frac{k(k-1)}{2}} \biggl(\frac{x}{b_{n}} \biggr)^{k} \\ &\quad\times\prod _{s=0}^{(n_{m}-k-1)} \biggl(p^{s}-q^{s} \frac{x}{b_{n}} \biggr)\bigg| f \biggl(\frac{p^{n_{m}-k}[k]_{p,q}+\alpha}{[n]_{p,q}+\beta}b_{n} \biggr)-f(x) \bigg| \\ \leq& M\frac{1}{p^{\frac{n_{m}(n_{m}-1)}{2}}}\sum_{k=0}^{n_{m}} \left [ \textstyle\begin{array}{c} n_{m} \\ k \end{array}\displaystyle \right ]_{p,q} p^{\frac{k(k-1)}{2}} \biggl(\frac{x}{b_{n}} \biggr)^{k}\\ &\quad\times\prod _{s=0}^{(n_{m}-k-1)} \biggl(p^{s}-q^{s} \frac{x}{b_{n}} \biggr)\bigg| \biggl(\frac{p^{n_{m}-k}[k]_{p,q}+\alpha}{[n]_{p,q}+\beta}b_{n} \biggr)-x \bigg|^{\gamma}. \end{aligned}$$

Using Hölder’s inequality with the values $p=\frac{2}{\gamma}$ and $q=\frac{2}{2-\gamma}$, we get

$$\begin{aligned} \big| C_{n,m}^{\alpha,\beta}(f;x,p,q)- f(x)\big| \leq{}&\frac{M}{p^{\frac{n_{m}(n_{m}-1)}{2}}} \sum_{k=0}^{n_{m}} \Biggl[ \Biggl\{ \left [ \textstyle\begin{array}{c} n_{m} \\ k \end{array}\displaystyle \right ]_{p,q} p^{\frac{k(k-1)}{2}} \biggl(\frac{x}{b_{n}} \biggr)^{k}\\ &\times\prod _{s=0}^{(n_{m}-k-1)} \biggl(p^{s}-q^{s} \frac{x}{b_{n}} \biggr) \biggl( \biggl(\frac{p^{n_{m}-k}[k]_{p,q}+\alpha}{[n]_{p,q}+\beta}b_{n} \biggr) -x \biggr)^{2} \Biggr\} ^{\frac{\gamma}{2}} \\ & \times \Biggl\{ \left [ \textstyle\begin{array}{c} n_{m} \\ k \end{array}\displaystyle \right ]_{p,q}p^{\frac{k(k-1)}{2}} \biggl(\frac{x}{b_{n}} \biggr)^{k} \prod_{s=0}^{(n_{m}-k-1)} \biggl(p^{s}-q^{s}\frac{x}{b_{n}} \biggr) \Biggr\} ^{\frac {2-\gamma}{2}} \Biggr] \\ \leq {} &M \Biggl[ \Biggl\{ \frac{1}{p^{\frac{n_{m}(n_{m}-1)}{2}}}\sum_{k=0}^{n_{m}} \left [ \textstyle\begin{array}{c} n_{m} \\ k \end{array}\displaystyle \right ]_{p,q} p^{\frac{k(k-1)}{2}} \biggl(\frac{x}{b_{n}} \biggr)^{k}\\ &\times \prod _{s=0}^{(n_{m}-k-1)} \biggl(p^{s}-q^{s} \frac{x}{b_{n}} \biggr) \biggl( \biggl(\frac{p^{n_{m}-k}[k]_{p,q}+\alpha}{[n]_{p,q}+\beta}b_{n} \biggr) -x \biggr)^{2} \Biggr\} ^{\frac{\gamma}{2}} \\ & \times \Biggl\{ \frac {1}{p^{\frac{n_{m}(n_{m}-1)}{2}}} \sum_{k=0}^{n_{m}} \left [ \textstyle\begin{array}{c} n_{m} \\ k \end{array}\displaystyle \right ]_{p,q}p^{\frac{k(k-1)}{2}} \biggl(\frac{x}{b_{n}} \biggr)^{k}\\ &\times \prod _{s=0}^{(n_{m}-k-1)} \biggl(p^{s}-q^{s} \frac{x}{b_{n}} \biggr) \Biggr\} ^{\frac {2-\gamma}{2}} \Biggr] \\ ={}& M \bigl[C_{n,m}^{\alpha,\beta}\bigl((t-x)^{2};x,p,q\bigr) \bigr]^{\frac{\gamma }{2}} \\ \leq{}& M \bigl(\lambda_{n,p,q}(x)\bigr)^{\frac{\gamma}{2}}. \end{aligned}$$

 □

In order to obtain rate of convergence in terms of modulus of continuity $\omega(f;\delta)$, we assume that, for any $f \in C_{B}[0,\infty)$ and $x \geq0$, the modulus of continuity of *f* is given by

3.7 $$ \omega(f;\delta)=\max_{\substack {|t-x|\leq\delta\\ t,x\in[0,\infty)}}\big|f(t)-f(x)\big|. $$

Thus it implies for any $\delta> 0$

3.8 $$ \big|f(x)-f(y)\big| \leq\omega(f;\delta) \biggl( \frac{|x-y|}{\delta}+1 \biggr). $$

#### Theorem 5

*If* $f \in C_{B}[0,\infty)$, *we have*

$$\big|C_{n,m}^{\alpha,\beta}(f;x,p,q)- f(x)\big| \leq2 \omega\bigl(f;\sqrt{ \lambda _{n,p,q}(x)}\bigr), $$

*where* $\omega(f;\cdot)$ *is the modulus of continuity of* *f* *and* $\lambda _{n,p,q}(x)$ *is the same as in Theorem* 4.

#### Proof

Using the triangular inequality, we get

$$\begin{aligned} \big|C_{n,m}^{\alpha,\beta}(f;x,p,q)- f(x)\big|={}& \Bigg|\frac{1}{p^{\frac{n_{m}(n_{m}-1)}{2}}}\sum _{k=0}^{n_{m}} \left [ \textstyle\begin{array}{c} n_{m} \\ k \end{array}\displaystyle \right ]_{p,q} p^{\frac{k(k-1)}{2}} \biggl( \frac{x}{b_{n}} \biggr)^{k}\\ &\times \prod_{s=0}^{(n_{m}-k-1)} \biggl(p^{s}-q^{s}\frac{x}{b_{n}} \biggr) f \biggl(\frac{p^{n_{m}-k}[k]_{p,q}+\alpha}{[n]_{p,q}+\beta}b_{n} \biggr)-f(x) \Bigg| \\ \leq{}& \frac{1}{p^{\frac{n_{m}(n_{m}-1)}{2}}}\sum_{k=0}^{n_{m}} \left [ \textstyle\begin{array}{c} n_{m} \\ k \end{array}\displaystyle \right ]_{p,q} p^{\frac{k(k-1)}{2}} \biggl(\frac{x}{b_{n}} \biggr)^{k}\\ &\times \prod _{s=0}^{(n_{m}-k-1)} \biggl(p^{s}-q^{s} \frac{x}{b_{n}} \biggr) \bigg| f \biggl(\frac{p^{n_{m}-k}[k]_{p,q}+\alpha}{[n]_{p,q}+\beta}b_{n} \biggr)-f(x) \bigg| . \end{aligned}$$

Now using (3.8) and Hölder’s inequality, we get

$$\begin{aligned} \big|C_{n,m}^{\alpha,\beta}(f;x,p,q)- f(x)\big| ={}& \frac{1}{p^{\frac{n_{m}(n_{m}-1)}{2}}}\sum_{k=0}^{n_{m}} \left [ \textstyle\begin{array}{c} n_{m} \\ k \end{array}\displaystyle \right ]_{p,q} p^{\frac{k(k-1)}{2}} \biggl(\frac{x}{b_{n}} \biggr)^{k}\\ &\times \prod _{s=0}^{(n_{m}-k-1)} \biggl(p^{s}-q^{s} \frac{x}{b_{n}} \biggr) \biggl( \frac{ \vert \frac{p^{n_{m}-k}[k]_{p,q}+\alpha}{[n]_{p,q}+\beta}b_{n}-x \vert }{\delta}+1 \biggr)\omega(f;\delta) \\ \leq{}&\omega(f;\delta) \frac{1}{p^{\frac{n_{m}(n_{m}-1)}{2}}}\sum_{k=0}^{n_{m}} \left [ \textstyle\begin{array}{c} n_{m} \\ k \end{array}\displaystyle \right ]_{p,q} p^{\frac{k(k-1)}{2}} \biggl(\frac{x}{b_{n}} \biggr)^{k}\\ &\times \prod _{s=0}^{(n_{m}-k-1)} \biggl(p^{s}-q^{s} \frac{x}{b_{n}} \biggr) \\ &+\frac{\omega(f;\delta)}{\delta} \frac{1}{p^{\frac{n_{m}(n_{m}-1)}{2}}}\sum _{k=0}^{n_{m}} \left [ \textstyle\begin{array}{c} n_{m} \\ k \end{array}\displaystyle \right ]_{p,q} p^{\frac{k(k-1)}{2}} \biggl(\frac{x}{b_{n}} \biggr)^{k}\\ &\times \prod_{s=0}^{(n_{m}-k-1)} \biggl(p^{s}-q^{s}\frac{x}{b_{n}} \biggr) \biggl\vert \frac {p^{n_{m}-k}[k]_{p,q}+\alpha}{[n]_{p,q}+\beta}b_{n}-x \biggr\vert \\ ={}& \omega(f;\delta)+\frac{\omega(f;\delta)}{\delta } \Biggl\{ \frac{1}{p^{\frac{n_{m}(n_{m}-1)}{2}}}\sum _{k=0}^{n_{m}} \left [ \textstyle\begin{array}{c} n_{m} \\ k \end{array}\displaystyle \right ]_{p,q} p^{\frac{k(k-1)}{2}} \biggl(\frac{x}{b_{n}} \biggr)^{k} \\ &\times\prod_{s=0}^{(n_{m}-k-1)} \biggl(p^{s}-q^{s}\frac{x}{b_{n}} \biggr) \biggl(\frac{p^{n_{m}-k}[k]_{p,q}+\alpha}{[n]_{p,q}+\beta}b_{n}-x \biggr)^{2} \Biggr\} ^{\frac{1}{2}} \\ ={}& \omega(f;\delta)+\frac{\omega(f;\delta)}{\delta } \bigl\{ C_{n,m}^{\alpha,\beta} \bigl((t-x)^{2};x,p,q\bigr) \bigr\} ^{1/2}. \end{aligned}$$

Now choosing $\delta=\lambda_{n,p,q}(x)$ as in Theorem 4, we have

$$\big|C_{n,m}^{\alpha,\beta}(f;x,p,q)- f(x)\big| \leq2 \omega\bigl(f;\sqrt{ \lambda _{n,p,q}(x)}\bigr). $$

 □

Next we calculate the rate of convergence in terms of the modulus of continuity of the derivative of a function.

#### Theorem 6

*Let* $A>0$. *If* $f(x)$ *has a continuous bounded derivative* $f {'}(x)$ *and* $\omega(f{'} ; \delta)$ *is the modulus of continuity of* $f{'}(x)$ *in* $x\in[0,\max\{b_{n},A\}]$, *then*

$$\begin{gathered} \big|f(x)-C_{n,m}^{\alpha,\beta}(f;x,p,q)\big| \\ \quad\leq M \biggl( \biggl\vert \frac{[n_{m}]_{p,q}}{[n]_{p,q}+\beta}-1 \biggr\vert A+ \frac {\alpha b_{n}}{[n]_{p,q}+\beta} \biggr)+2\bigl(B_{n,p,q}(\alpha,\beta) \bigr)^{1/2} \omega\bigl(f{'};\bigl(B_{n,p,q}(\alpha, \beta)\bigr)^{1/2}\bigr), \end{gathered}$$

*where* *M* *is a positive constant such that* $|f{'}(x)| \leq M$ *and*

$$\begin{aligned} B_{n,p,q}(\alpha,\beta)={}& \bigg|1-2\frac{[n_{m}]_{p,q}}{([n]_{p,q}+\beta )}+\frac{q[n_{m}]_{p,q}[n_{m}-1]_{p,q}}{([n]_{p,q}+\beta)^{2}} \bigg|A^{2} \\ &+ \biggl\vert \frac{[n_{m}]_{p,q}(2\alpha+p^{n_{m}-1})}{([n]_{p,q}+\beta )^{2}}-\frac{2 \alpha}{([n]_{p,q}+\beta)} \biggr\vert {A b_{n}} +\frac{\alpha^{2} b_{n}^{2}}{([n]_{p,q}+\beta)^{2}}. \end{aligned}$$

#### Proof

Using the mean value theorem, we have

$$\begin{gathered} f \biggl(\frac{p^{n_{m}-k}[k]_{p,q}+\alpha}{[n]_{p,q}+\beta}b_{n} \biggr)-f(x)\\ \quad = \biggl( \frac{p^{n_{m}-k}[k]_{p,q}+\alpha}{[n]_{p,q}+\beta }b_{n}-x \biggr) f{'}(\xi) \\ \quad = \biggl(\frac{p^{n_{m}-k}[k]_{p,q}+\alpha}{[n]_{p,q}+\beta }b_{n}-x \biggr)f{'}(x)+ \biggl(\frac{p^{n_{m}-k}[k]_{p,q}+\alpha }{[n]_{p,q}+\beta}b_{n}-x \biggr) \bigl(f{'}( \xi)-f{'}(x)\bigr), \end{gathered}$$

where *ξ* is a point between *x* and $\frac{p^{n_{m}-k}[k]_{p,q}+\alpha }{[n]_{p,q}+\beta}b_{n}$. By using the above identity, we get

$$\begin{aligned} C_{n,m}^{\alpha,\beta}(f;x,p,q)-f(x) ={}&f{'}(x) \frac{1}{p^{\frac{n_{m}(n_{m}-1)}{2}}}\sum_{k=0}^{n_{m}} \left [ \textstyle\begin{array}{c} n_{m} \\ k \end{array}\displaystyle \right ]_{p,q} p^{\frac{k(k-1)}{2}} \biggl(\frac{x}{b_{n}} \biggr)^{k}\\ &\times \prod _{s=0}^{(n_{m}-k-1)} \biggl(p^{s}-q^{s} \frac{x}{b_{n}} \biggr) \biggl(\frac {p^{n_{m}-k}[k]_{p,q}+\alpha}{[n]_{p,q}+\beta}b_{n}-x \biggr) \\ & +\frac {1}{p^{\frac{n_{m}(n_{m}-1)}{2}}}\sum_{k=0}^{n_{m}} \left [ \textstyle\begin{array}{c} n_{m} \\ k \end{array}\displaystyle \right ]_{p,q} p^{\frac{k(k-1)}{2}} \biggl(\frac{x}{b_{n}} \biggr)^{k}\\ &\times \prod _{s=0}^{(n_{m}-k-1)} \biggl(p^{s}-q^{s} \frac{x}{b_{n}} \biggr) \biggl(\frac {p^{n_{m}-k}[k]_{p,q}+\alpha}{[n]_{p,q}+\beta}b_{n}-x \biggr) \bigl(f{'}(\xi)-f{'}(x)\bigr). \end{aligned}$$

Hence,

$$\begin{aligned} \big|C_{n,m}^{\alpha,\beta}(f;x,p,q)-f(x)\big| \leq{}&\big|f{'}(x)\big| \cdot \big|C_{n,m}^{\alpha,\beta}\bigl((t-x);x,p,q\bigr)\big| \\ & + \frac{1}{p^{\frac{n_{m}(n_{m}-1)}{2}}}\sum_{k=0}^{n_{m}} \left [ \textstyle\begin{array}{c} n_{m} \\ k \end{array}\displaystyle \right ]_{p,q} p^{\frac{k(k-1)}{2}} \biggl(\frac{x}{b_{n}} \biggr)^{k}\\ &\times \prod _{s=0}^{(n_{m}-k-1)} \biggl(p^{s}-q^{s} \frac{x}{b_{n}} \biggr) \biggl\vert \frac {p^{n_{m}-k}[k]_{p,q}+\alpha}{[n]_{p,q}+\beta}b_{n}-x \biggr\vert \big|f{'}(\xi )-f{'}(x)\big| \\ \leq{}& M \biggl( \biggl\vert \frac {[n_{m}]_{p,q}}{[n]_{p,q}+\beta}-1 \biggr\vert A+ \frac{\alpha b_{n}}{[n]_{p,q}+\beta} \biggr) \\ & + \frac{1}{p^{\frac{n_{m}(n_{m}-1)}{2}}}\sum_{k=0}^{n_{m}} \left [ \textstyle\begin{array}{c} n_{m} \\ k \end{array}\displaystyle \right ]_{p,q} p^{\frac{k(k-1)}{2}} \biggl(\frac{x}{b_{n}} \biggr)^{k} \\ &\times\prod _{s=0}^{(n_{m}-k-1)} \biggl(p^{s}-q^{s} \frac{x}{b_{n}} \biggr) \biggl\vert \frac {p^{n_{m}-k}[k]_{p,q}+\alpha}{[n]_{p,q}+\beta}b_{n}-x \biggr\vert \big|f{'}(\xi )-f{'}(x)\big| \\ \leq{}& M \biggl( \biggl\vert \frac{[n+m]_{p,q}}{[n]_{p,q}+\beta}-1 \biggr\vert A+ \frac {\alpha b_{n}}{[n]_{p,q}+\beta} \biggr) \\ & + \frac{1}{p^{\frac{n_{m}(n_{m}-1)}{2}}}\sum_{k=0}^{n_{m}} \left [ \textstyle\begin{array}{c} n_{m} \\ k \end{array}\displaystyle \right ]_{p,q} p^{\frac{k(k-1)}{2}} \biggl(\frac{x}{b_{n}} \biggr)^{k}\\ &\times \prod _{s=0}^{(n_{m}-k-1)} \biggl(p^{s}-q^{s} \frac{x}{b_{n}} \biggr) \biggl\vert \frac {p^{n_{m}-k}[k]_{p,q}+\alpha}{[n]_{p,q}+\beta}b_{n}-x \biggr\vert \\ & \times\omega\bigl(f{'};\delta\bigr) \biggl( \frac{ \vert \frac {p^{n_{m}-k}[k]_{p,q}+\alpha}{[n]_{p,q}+\beta}b_{n}-x \vert }{\delta}+1 \biggr), \end{aligned}$$

since

$$ |\xi-x| \leq \biggl\vert \frac{p^{n_{m}-k}[k]_{p,q}+\alpha}{[n]_{p,q}+\beta }b_{n}-x \biggr\vert . $$

Using it, we have

$$\begin{aligned} \big|C_{n,m}^{\alpha,\beta}(f;x,p,q)-f(x)\big| \leq {}&M \biggl( \biggl\vert \frac{[n_{m}]_{p,q}}{[n]_{p,q}+\beta}-1 \biggr\vert A+ \frac{\alpha b_{n}}{[n]_{p,q}+\beta} \biggr) \\ & + \omega\bigl(f{'};\delta\bigr)\frac{1}{p^{\frac{n_{m}(n_{m}-1)}{2}}}\sum _{k=0}^{n_{m}} \left [ \textstyle\begin{array}{c} n_{m} \\ k \end{array}\displaystyle \right ]_{p,q} p^{\frac{k(k-1)}{2}} \biggl(\frac{x}{b_{n}} \biggr)^{k}\\ &\times \prod_{s=0}^{(n_{m}-k-1)} \biggl(p^{s}-q^{s}\frac{x}{b_{n}} \biggr) \biggl\vert \frac {p^{n_{m}-k}[k]_{p,q}+\alpha}{[n]_{p,q}+\beta}b_{n}-x \biggr\vert \\ & + \frac{\omega(f{'};\delta)}{\delta}\frac{1}{p^{\frac{n_{m}(n_{m}-1)}{2}}}\sum_{k=0}^{n_{m}} \left [ \textstyle\begin{array}{c} n_{m} \\ k \end{array}\displaystyle \right ]_{p,q} p^{\frac{k(k-1)}{2}} \biggl(\frac{x}{b_{n}} \biggr)^{k}\\ &\times \prod _{s=0}^{(n_{m}-k-1)} \biggl(p^{s}-q^{s} \frac{x}{b_{n}} \biggr) \biggl\vert \frac {p^{n_{m}-k}[k]_{p,q}+\alpha}{[n]_{p,q}+\beta}b_{n}-x \biggr\vert ^{2}. \end{aligned}$$

Now using Cauchy-Schwarz inequality for the second term, we obtain

$$\begin{aligned} \big|C_{n,m}^{\alpha,\beta}(f;x,p,q)-f(x)\big| \leq{}& M \biggl( \biggl\vert \frac{[n_{m}]_{p,q}}{[n]_{p,q}+\beta}-1 \biggr\vert A+ \frac{\alpha b_{n}}{[n]_{p,q}+\beta} \biggr) \\ & + \omega\bigl(f{'};\delta\bigr) \Biggl(\frac{1}{p^{\frac{n_{m}(n_{m}-1)}{2}}}\sum _{k=0}^{n_{m}} \left [ \textstyle\begin{array}{c} n_{m} \\ k \end{array}\displaystyle \right ]_{p,q} p^{\frac{k(k-1)}{2}} \biggl( \frac{x}{b_{n}} \biggr)^{k}\\ &\times \prod_{s=0}^{(n_{m}-k-1)} \biggl(p^{s}-q^{s}\frac{x}{b_{n}} \biggr) \biggl\vert \frac {p^{n_{m}-k}[k]_{p,q}+\alpha}{[n]_{p,q}+\beta}b_{n}-x \biggr\vert ^{2} \Biggr)^{1/2} \\ & + \frac{\omega(f{'};\delta)}{\delta}\frac{1}{p^{\frac{n_{m}(n_{m}-1)}{2}}}\sum_{k=0}^{n_{m}} \left [ \textstyle\begin{array}{c} n_{m} \\ k \end{array}\displaystyle \right ]_{p,q} p^{\frac{k(k-1)}{2}} \biggl(\frac{x}{b_{n}} \biggr)^{k}\\ &\times \prod _{s=0}^{(n_{m}-k-1)} \biggl(p^{s}-q^{s} \frac{x}{b_{n}} \biggr) \biggl\vert \frac {p^{n_{m}-k}[k]_{p,q}+\alpha}{[n]_{p,q}+\beta}b_{n}-x \biggr\vert ^{2} \\ ={}&M \biggl( \biggl\vert \frac{[n_{m}]_{p,q}}{[n]_{p,q}+\beta}-1 \biggr\vert A+ \frac{\alpha b_{n}}{[n]_{p,q}+\beta} \biggr) \\ & + \omega\bigl(f{'};\delta\bigr)\sqrt{C_{n,m}^{\alpha,\beta} \bigl((t-x)^{2};x,p,q\bigr)}\\ &+\frac {\omega(f{'};\delta)}{\delta}C_{n,m}^{\alpha,\beta} \bigl((t-x)^{2};x,p,q\bigr). \end{aligned}$$

Using Lemma 2, we see

$$\begin{aligned} \sup_{0\leq x \leq A} C_{n,m}^{(\alpha,\beta)} \bigl((t-x)^{2};x,p,q\bigr) \leq{}&\sup_{0\leq x \leq A} \biggl[ \biggl(1-2\frac {[n_{m}]_{p,q}}{([n]_{p,q}+\beta)}+ \frac {q[n_{m}]_{p,q}[n_{m}-1]_{p,q}}{([n]_{p,q}+\beta)^{2}} \biggr)x^{2}\\ & + \biggl( \frac{[n_{m}]_{p,q}(2\alpha+p^{n_{m}-1})}{([n]_{p,q}+\beta)}-2 \alpha \biggr) \frac{b_{n}x}{([n]_{p,q}+\beta)}\\ &+\frac{\alpha^{2} b_{n}^{2}}{([n]_{p,q}+\beta)^{2}} \biggr] \\ \leq{}& \bigg|1-2\frac{[n_{m}]_{p,q}}{([n]_{p,q}+\beta)}+\frac {q[n_{m}]_{p,q}[n_{m}-1]_{p,q}}{([n]_{p,q}+\beta)^{2}} \bigg|A^{2}\\ & + \biggl\vert \frac{[n_{m}]_{p,q}(2\alpha+p^{n_{m}-1})}{([n]_{p,q}+\beta )^{2}}-\frac{2 \alpha}{([n]_{p,q}+\beta)} \biggr\vert {A b_{n} } \\&+\frac{\alpha^{2} b_{n}^{2}}{([n]_{p,q}+\beta)^{2}} \\ :={}&B_{n,p,q}(\alpha ,\beta). \end{aligned}$$

Thus,

$$\begin{aligned} \big|C_{n,m}^{\alpha,\beta}(f;x,p,q)-f(x)\big| \leq{}& M \biggl( \biggl\vert \frac {[n_{m}]_{p,q}}{[n]_{p,q}+\beta}-1 \biggr\vert A+\frac{\alpha b_{n}}{[n]_{p,q}+\beta} \biggr)\\&+ \omega \bigl(f{'};\delta\bigr) \biggl[ \bigl(B_{n,p,q}(\alpha,\beta) \bigr)^{1/2}+\frac {1}{\delta}B_{n,p,q}(\alpha,\beta) \biggr]. \end{aligned}$$

Choosing $\delta:=(B_{n,p,q}(\alpha,\beta))^{1/2} $, we get the desired result. □

### Voronovskaja-type result

Now, we prove a Voronovskaja-type approximation theorem with the help of the $C_{n,m}^{(\alpha,\beta)}$ family of linear operators defined by (2.1).

#### Lemma 4

*Let* $(p_{n})$ *and* $(q_{n})$ *be two sequences satisfying* (3.2) *and* $x \in[0, E]$ *where* $E \in\mathbb {R}^{+}$. *Then we get*

3.9 $$\begin{aligned} \lim_{n \to\infty}\frac{[n]_{p_{n},q_{n}}}{b_{n}} C_{n,m}^{(\alpha ,\beta)} (t-x; x,p_{n},q_{n} )=\alpha \end{aligned}$$

*and*

3.10 $$\begin{aligned} \lim_{n\to\infty}\frac{[n]_{p_{n},q_{n}}}{b_{n}} C_{n,m}^{(\alpha ,\beta)} \bigl((t-x)^{2}; x,p_{n},q_{n} \bigr)= ax, \end{aligned}$$

*where* $a \in(0, 1)$.

#### Proof

We shall prove only (3.10) because the proof of (3.9) is similar. Let $x \in[0, E]$. Then, by Lemma (2), we obtain, for all $n \in\mathbb {N}$,

3.11 $$ \begin{aligned}[b]& \frac{[n]_{p_{n},q_{n}}}{b_{n}}C_{n,m}^{(\alpha,\beta )} \bigl((t-x)^{2};x,p_{n},q_{n}\bigr)\\ &\quad= \frac{[n]_{p_{n},q_{n}}}{b_{n}} \biggl(1-2\frac {[n_{m}]_{p_{n},q_{n}}}{([n]_{p_{n},q_{n}}+\beta)}+\frac {q_{n}[n_{m}]_{p_{n},q_{n}}[n_{m}-1]_{p_{n},q_{n}}}{([n]_{p_{n},q_{n}}+\beta)^{2}} \biggr)x^{2} \\ &\qquad + \biggl( \frac{(2\alpha+p_{n}^{n-1})[n_{m}]_{p_{n},q_{n}}}{([n]_{p_{n},q_{n}}+\beta )}-2\alpha \biggr) \frac{[n]_{p_{n},q_{n}}}{([n]_{p_{n},q_{n}}+\beta)} x + \frac {\alpha^{2} b_{n} [n]_{p_{n},q_{n}}}{([n]_{p_{n},q_{n}}+\beta)^{2}}. \end{aligned}$$

Now by taking the limit as $n \to\infty$ in (3.11), we obtain

$$ \lim_{n\to\infty}\frac{[n]_{p_{n},q_{n}}}{b_{n}}C_{n,m}^{(\alpha,\beta )} \bigl((t-x)^{2};x,p_{n},q_{n}\bigr)= ax, $$

which completes the proof. □

In a similar way to Lemma 4 one can deduce the following lemma.

#### Lemma 5

*Let* $(p_{n})$ *and* $(q_{n})$ *be two sequences satisfying* (3.2) *and* $x \in[0, E]$ *where* $E \in\mathbb {R}^{+}$. *There is a positive constants* $M_{0}(x)$ *depending only on* *x* *such that*

3.12 $$ \lim_{n}\frac{[n]^{2}_{p_{n},q_{n}}}{b^{2}_{n}} C_{n,m}^{(\alpha,\beta)} \bigl((t-x)^{4}; x,p_{n},q_{n} \bigr)\leq M_{0}(x). $$

#### Theorem 7

*Let* $(p_{n})$ *and* $(q_{n})$ *be two sequences with the property* (3.2). *For every* $f\in C_{1+x^{2}}^{0}[0, \infty)$ *such that* $f',f'' \in C_{1+x^{2}}^{0}[0, \infty)$, *then*

$$\begin{aligned} \lim_{n\to\infty} \frac{[n]_{p_{n},q_{n}}}{b_{n}} \bigl[C_{n,m}^{(\alpha,\beta )} \bigl(f(t); x,p_{n},q_{n}\bigr)-f(x) \bigr] =\alpha f'(x)+\frac{1}{2}axf''(x) \end{aligned}$$

*uniformly in* $x \in[0, E]$.

#### Proof

Using the Taylor formula for $f\in C_{1+x^{2}}^{0}$, we have

$$ f(t)=f(x)+f'(x) (t-x)+\frac{1}{2}f''(x) (t-x)^{2}+\eta_{x}(t) (t-x)^{2}, $$

where the function $\eta_{x}(\cdot)$ is the remainder, $\lim_{t\to x}\eta_{x}(t)=0$. Since the operator $C_{n,m}^{(\alpha,\beta)}$ is linear

3.13 $$ \begin{aligned}[b] \frac{[n]_{p_{n},q_{n}}}{b_{n}} \bigl[C_{n,m}^{(\alpha,\beta )} \bigl(f(t); x,p_{n},q_{n}\bigr)-f(x) \bigr] ={}&\frac{[n]_{p_{n},q_{n}}}{b_{n}}f'(x)C_{n,m}^{(\alpha,\beta)}(t-x; x,p_{n},q_{n})\\ &+\frac{1}{2}\frac{[n]_{p_{n},q_{n}}}{b_{n}}f''(x)C_{n,m}^{(\alpha ,\beta)} \bigl((t-x)^{2}; x,p_{n},q_{n}\bigr) \\ &+\frac{[n]_{p_{n},q_{n}}}{b_{n}}C_{n,m}^{(\alpha,\beta)}\bigl(\eta_{x}(t) (t-x)^{2};x,p_{n},q_{n}\bigr) \end{aligned}$$

for each $n \in\mathbb {N}$. We will now show that

3.14 $$\begin{aligned} \lim_{n\to\infty}\frac{[n]_{p_{n},q_{n}}}{b_{n}}C_{n,m}^{(\alpha,\beta)} \bigl(\eta _{x}(t) (t-x)^{2};x,p_{n},q_{n} \bigr)=0. \end{aligned}$$

After application of the Cauchy-Schwarz inequality for the third term on the right hand side of (3.13), we find that

3.15 $$ \begin{aligned}[b] &\frac{[n]_{p_{n},q_{n}}}{b_{n}}C_{n,m}^{(\alpha,\beta)} \bigl(\eta _{x}(t) (t-x)^{2};x,p_{n},q_{n} \bigr) \\ &\quad\leq \frac{[n]_{p_{n},q_{n}}}{b_{n}}\bigl[C_{n,m}^{(\alpha,\beta)}\bigl(\eta ^{2}_{x}(t);x,p_{n},q_{n}\bigr) \bigr]^{1/2}\bigl[C_{n,m}^{(\alpha,\beta)}\bigl((t-x)^{4};x,p_{n},q_{n} \bigr)\bigr]^{1/2}. \end{aligned}$$

Let us take $\eta^{2}_{x}(t)=\theta_{x}(t)$, $x \geq0$, we obtain

$$\begin{aligned} &\lim_{|x| \to\infty} \frac{|\theta_{x}(t)|}{1+x^{2}} =\lim_{|x| \to\infty} \frac{|f(t)-f(x)-f'(x)(t-x)-\frac {1}{2}f''(x)(t-x)^{2}|^{2}}{(t-x)^{4} (1+x^{2})}. \end{aligned}$$

We have $f \in C_{1+x^{2}}^{0}$ *i.e.* $\lim_{|x| \to\infty} \frac{|f(x)|}{1+x^{2}} =$ finite value, which means *f* is function with maximum order of *x* is 2. Henceforth *x* is of order 1 and 0, respectively, in $f{'}$ and $f{''}$, *i.e.* $f{''}$ is constant.

We will get a finite value of the above limit because numerator is a polynomial in *x* having terms of degree less than or equal to four and $f,f',f'' \in C_{1+x^{2}}^{0}$. Thus $\theta_{x}(t) \in C_{1+x^{2}}^{0}$.

Moreover, $\lim_{t\to x}\theta_{x}(t)=0$. From Theorem 2, we observe that

3.16 $$\begin{aligned} \lim_{n\to\infty}C_{n,m}^{(\alpha,\beta)}\bigl( \eta^{2}_{x}(t);x,p_{n},q_{n}\bigr)=\lim _{n\to\infty}C_{n,m}^{(\alpha,\beta)}\bigl(\theta_{x}(t);x,p_{n},q_{n} \bigr)=\theta_{x}(x)=0 \end{aligned}$$

uniformly in $x \in[0, E]$. One obtains from Lemma 5 that

3.17 $$\begin{aligned} \lim_{n \to\infty}\frac{[n]^{2}_{p_{n},q_{n}}}{b^{2}_{n}} C_{n,m}^{(\alpha,\beta )} \bigl((t-x)^{4}; x,p_{n},q_{n} \bigr)\leq M_{0}(x). \end{aligned}$$

From these last two relations, the inclusion (3.14) holds true. Now by taking the limit as $n \to\infty$ in (3.13) and using Lemma (4), we conclude that

$$\begin{aligned} \lim_{n\to\infty} \frac{[n]_{p_{n},q_{n}}}{b_{n}} \bigl[C_{n,m}^{(\alpha,\beta )}\bigl(f(t); x,p_{n},q_{n} \bigr)-f(x) \bigr] =\alpha f'(x)+\frac{1}{2}axf''(x) \end{aligned}$$

uniformly in $x \in[0, E]$, which leads us to the desired assertion of Theorem 7. □

### Example

With the help of Maple, we show a comparison of the $(p,q)$ Bernstein-stancu operator and the operator (2.1) to the function $f(x) = \sin(x)$ under the following parameters: $\alpha= 1$, $\beta= 1$, $p = 0.9$, $q = 0.8$, $n = 1$ and $b_{n}=\ln(1+n)$ within the interval $[0,b_{1}]$ *i.e.* $[0,\log_{e} 11]$. We have found it to be convenient to investigate our series only for finite sums. More powerful equipments with higher speed can easily compute the more complicated infinite series in a similar manner.

**Figure 1:**
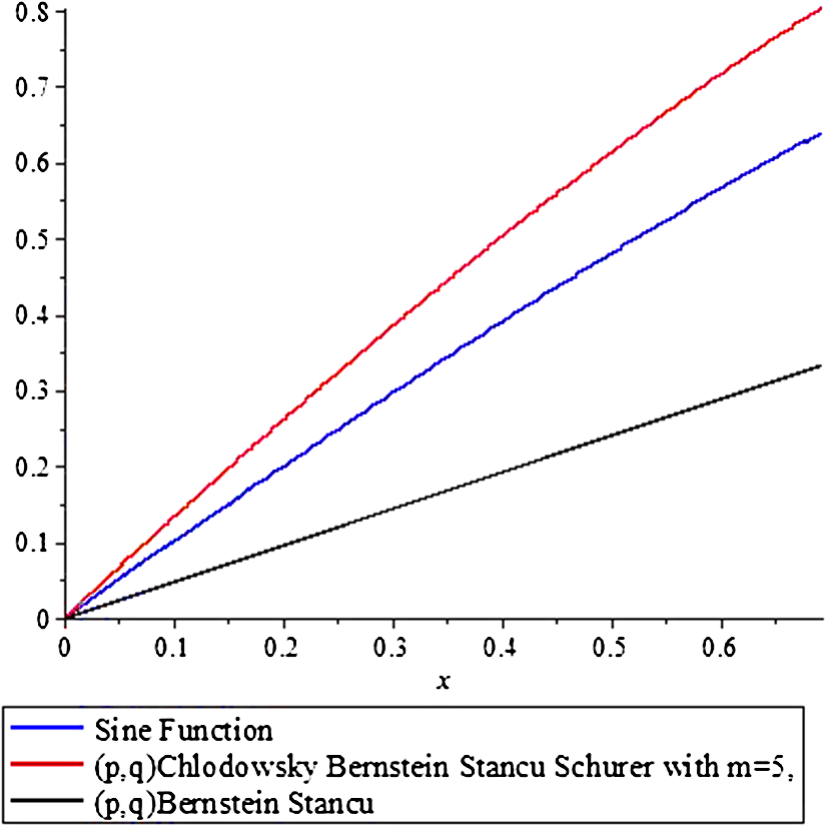
**Comparison of** $\pmb{(p,q)}$ **Chlodowsky type Bernstein-Stancu-Schurer operators and** $\pmb{(p,q)}$ **Bernstein-Stancu operators for** $\pmb{\operatorname{Sin}(x)}$ **.**

It is clear from the Figure 1 that approximation by the operator (2.1) is better than by $(p,q)$ Bernstein-stancu operator for $f(x)=\sin x$ and it can be improved further by taking appropriate values of *m* and sequence $b_{n}$.

## Conclusion

A better approximation of complex functions over the required interval $[0,b_{n}]$ can be attained using the Chlodowsky variant of the $(p,q)$ Bernstein-Stancu-Schurer operator for choosing suitable values of the sequence $b_{n}$ and *n* compared to classical operators over the fixed interval $[0,1]$.
